# Effect of irrigant type, activation method, and contact time on dentin diffusion and ultrastructure

**DOI:** 10.1590/1807-3107bor-2026.vol40.084

**Published:** 2026-10-26

**Authors:** Vanessa Abreu Sanches Marques Costa, Letycia Accioly Simões Coelho, Cláudia Cristina Biguetti, Leticia Citelli Conti, Angélica Cristina Fonseca, Kelly Christian Tolentino Dominguez, Carla Regina Albino, Luciano Bachmann, Marco Antonio Hungaro Duarte, Rodrigo Ricci Vivan

**Affiliations:** a Universidade de São Paulo – USP, Bauru School of Dentistry, Department of Dentistry, Endodontics, and Dental Materials, Bauru, SP, Brazil.; b University of Texas at Rio Grande Valley (UTRGV), School of Podiatric Medicine, Rio Grande Valley, TX, USA.; c Universidade de São Paulo – USP, Bauru School of Dentistry, Department of Biological Sciences, Bauru, SP, Brazil.; d Universidade Estadual da Paraíba – UEPB, Department of Physics, Araruna, PB, Brazil.; e Universidade de São Paulo – USP, School of Philosophy, Sciences and Letters of Ribeirão Preto, Ribeirão Preto, SP, Brazil.

**Keywords:** Dentin, Sodium Hypochlorite, Chlorhexidine, Collagen, Tomography, Optical Coherence

## Abstract

This study aimed to evaluate the effects of different endodontic irrigation protocols on irrigant diffusion, dentin microhardness, and dentinal collagen organization. Bovine dentin specimens were irrigated with 2.5% sodium hypochlorite (NaOCl), 2% chlorhexidine (CHX), or 0.9% saline solution (SS) and subdivided according to the irrigation protocol as follows: conventional irrigation (CI), passive ultrasonic irrigation (PUI), Easy Clean activation (EC), and prolonged contact without activation. Irrigant diffusion within dentin was evaluated by optical coherence tomography (OCT), and dentin microhardness was measured using Knoop microhardness testing before and after the irrigation protocols. Dentinal collagen organization was assessed by Picrosirius Red staining under polarized light microscopy. Data were analyzed using nonparametric tests with a significance level of 5%. OCT analysis showed no significant differences in irrigant diffusion among NaOCl and CHX across the irrigation protocols tested (p>0.05). Dentin microhardness values did not differ significantly before and after irrigation for any solution or protocol tested (p > 0.05). Picrosirius Red analysis showed no significant differences in total birefringent area among groups (p>0.05). However, prolonged exposure to chlorhexidine resulted in significant differences in the distribution of collagen fiber birefringence for yellow, red, and green fibers compared with other irrigation protocols (p < 0.05). The irrigation protocols evaluated did not affect dentin microhardness or irrigant diffusion, but influenced dentinal collagen organization and surface morphology in an irrigant- and exposure time-dependent manner.

## Introduction

Irrigation is a critical step in endodontic treatment to address the anatomical complexity of the root canal system, particularly in regions that remain inaccessible to instruments.^1,2^ As irrigating solutions come into direct contact with dentin surfaces throughout the procedure, their chemical properties and application protocols may influence the structural and mechanical characteristics of dentin.^3,4^

Sodium hypochlorite and chlorhexidine are widely used irrigation solutions in endodontic therapy because of their well-established antimicrobial properties and broad clinical applicability.^5,6^ Sodium hypochlorite is considered the primary irrigant due to its unique ability to dissolve organic tissue in addition to its antimicrobial activity.^1^ However, several studies have reported that sodium hypochlorite induces structural alterations in dentin, with the extent of these effects depending on factors such as irrigant concentration, contact time, volume, and the method of irrigant delivery and activation.^2^

Conventional syringe-and-needle irrigation has limitations related to the vapor lock effect, which restricts irrigant exchange and penetration in anatomically complex regions of the root canal system.^7^ To overcome these limitations, irrigant activation techniques have been proposed. Passive ultrasonic irrigation has been widely investigated and enhances irrigant movement through acoustic streaming and cavitation, increasing irrigant penetration and interaction with dentin surfaces.^8,9^ Mechanical agitation devices, such as Easy Clean (Easy Equipamentos Odontológicos, Minas Gerais, Brazil), have emerged as alternative activation approaches, using rotary or reciprocating motion to agitate the irrigant and improve its contact with canal walls.^10^

However, the interaction among irrigation solutions, activation protocols, and dentin may alter the its organic matrix and mechanical properties.^11^ Therefore, careful selection of irrigant type and concentration, exposure time, and activation protocol is essential to balance effective disinfection with preservation of dentin structure.^12^

Optical coherence tomography (OCT) is a noninvasive, high-resolution method of evaluation of dentin surface and subsurface microstructure, allowing detection of morphological alterations induced by irrigation protocols in the organic and inorganic matrix.^13,14^ Picrosirius Red staining under polarized light allows assessment of collagen fiber organization within the organic dentin matrix,^15^ whereas alterations in the inorganic phase are indirectly reflected by changes in dentin microhardness.^16^ Most previous studies have assessed irrigant-induced dentin alterations through isolated outcomes, which limits the interpretation of how structural, organic, and mineral changes relate to one another. By combining these three complementary methods, this study provides a multiparametric evaluation of how irrigation protocols affect dentin as a composite tissue.

Therefore, the aim of this study was to evaluate the influence of contact time and activation of 2.5% sodium hypochlorite and 2% chlorhexidine on irrigant diffusion, assessed by OCT, and on dentin ultrastructure, assessed through microhardness testing and polarized light microscopy with Picrosirius Red staining. The null hypothesis was that the evaluated parameters are not influenced by the irrigant type or irrigation protocol.

## Methods

This study was approved by the Animal Research Ethics Committee (protocol nº: 003/2018, March 2018) and was conducted following the Declaration of Helsinki.

### Sample size Calculation

Sample size was calculated using G*Power software v3.1 (Heinrich Heine University Düsseldorf, Germany), applying the Wilcoxon-Mann-Whitney test. The effect size was set at 1.20, with an alpha error probability of 0.05, statistical power (1–β) of 0.80, and an allocation ratio (N2/N1) of 1. Based on these parameters, a minimum of five specimens per group was required to detect statistically significant differences.

### Sample preparation

A total of 45 single-rooted bovine teeth with intact crowns and roots were used. From the bovine crowns, 45 dentin slices (3×3×0.8 mm; length × width × thickness)^17^ were obtained using a precision cutting machine (Isomet 1000; Buehler Ltd., Lake Bluff, USA). From the cervical third of the roots, 90 dentin blocks (4.0 mm diameter; two blocks per tooth) were prepared using a trephine bur under copious water irrigation.

To better replicate clinical conditions, eighteen bovine incisor roots were selected, decoronated, standardized to a root length of 12 mm, and instrumented to size 40/.06 (MK Life, Porto Alegre, Brazil). In nine roots, a standardized cavity (3.5×3.5 mm) was prepared 3 mm from the apical foramen to accommodate the dentin slices used for OCT analysis. In the remaining nine roots, two standardized cavities were prepared to accommodate one dentin block for Picrosirius Red analysis and one dentin block for microhardness analysis.^18^ The dentin blocks and slices were fixed in the prepared cavities using a gingival barrier (FGM; Joinville, SC, Brazil), after which the irrigation protocols were applied. Each root was reused up to five times.

### Group allocation and irrigation protocol

The experimental groups were defined according to the irrigating solution: 0.9% saline solution (SS), 2.5% sodium hypochlorite (NaOCl) (Fórmula e Ação, São Paulo, Brazil), or 2% chlorhexidine digluconate (CHX) (Biodinâmica, Ibiporã, Brazil). All root canals were irrigated using a 5-mL syringe coupled to a 30-gauge needle (NaviTip; Ultradent Products Inc., South Jordan, USA) for 5 minutes, with simultaneous aspiration at a flow rate of approximately 1.2 mL/min, resulting in a total irrigant volume of 6 mL per tooth.

Four irrigation protocols were evaluated:

- NA (no activation): immersion in the irrigation solution for 30 min.
- CI (conventional irrigation): continuous syringe irrigation with simultaneous aspiration.
- UA (ultrasonic activation): irrigation was performed using an ultrasonic device (PM100 EMS; E.M.S. Electro Medical Systems S.A., Nyon, Switzerland) set at power level 1, coupled with an Irrisonic E1 insert (Helse Ultrasonic, São Paulo, SP, Brazil). The insert was positioned 2 mm short of the apical foramen and activated with vertical buccolingual movements for three 20-second cycles.^20^ The irrigating solution was renewed after each cycle.
- EA (EasyClean): agitation was performed using an EasyClean 25/.04 instrument (Easy Equipamentos Odontológicos, Minas Gerais, Brazil) attached to a contra-angle handpiece and operated with a micromotor at 20,000 rpm (KaVo Kerr Group, Charlotte, NC, USA). The instrument was positioned 2 mm short of the apical foramen and activated for three 20-second cycles,^20^ with solution replacement between cycles.

After irrigation, the specimens were transferred to microtubes containing 1.5 mL of distilled water and rinsed for 1 minute, including 15 seconds of ultrasonic agitation.

### Optical coherence tomography (OCT)

Before treatments, one surface of each dentin slice was polished under water with 4,000 grit silicon carbide abrasive paper (Buehler) followed by alumina suspensions of 1.0, 0.3, and 0.05 μm (Struers, Ballerup, Denmark) until a flat and smooth surface was obtained. Between each polishing step, specimens were immersed in distilled water to remove residual abrasives. The samples were then gently dried with absorbent paper, placed in a Petri dish, fixed on an XY translation stage, and covered with distilled water^21^

Structural characterization was perform.ed before and after the treatments using a spectral domain OCT system (OCP930SR; Thorlabs Inc., New Jersey, USA) equipped with a superluminescent light emitting diode (SLED) operating at 930 nm, with an output power of 2 mW and an axial resolution of 6.2 μm. The system provided a maximum imaging depth of 1.6 mm and a lateral scanning range of up to 6.0 mm, acquiring up to eight images per second^21^. OCT images were obtained using a Michelson interferometer-based system. Light was guided through an optical fiber to a 2×2 coupler, where It was divided into two beams: one directed to a reference mirror and the other to the sample. Light scattering was measured based on the proportional relationship between the reduction in light penetration and the density and thickness of the specimen (Lambert-Beer Law), resulting in the visualization of distinct layers with different grayscale intensities within the specimen.

Cross-sectional OCT images were acquired before and after the irrigation protocols from one margin of the sample to the other, totaling 800 frames per scan. This procedure provided a complete internal mapping. Three fields, starting from the center of the sample, were initially selected using MatLab software (version R2019a; MathWorks, Natick, USA). In all images, the polished surface interface was not measured, and the same region was delimited across all specimens (25 μm horizontal × 12 μm depth), to analyze the grayscale-level shift that occurs after irrigant application. The mean diffusion intensity value was measured using Zen 2.3 lite software to define the corresponding reflection area (μm) of the irrigants across the different protocols. All images were acquired by a single examiner blinded to group allocation.

### Microhardness analysis

Before the initial analysis, dentin surfaces were flattened and polished sequentially with 600, 800, and 1,200 grit silicon carbide papers (Extec Corp., Enfield, USA) under running water using a polishing machine (Politriz APL 4; Arotec, Cotia, Brazil). Between each polishing step, the specimens were cleaned with ultrasound for 2 minutes (Ultrasonic Cleaner USC 750; Unique Indústria e Comércio de Produtos Eletrônicos Ltda., São Paulo, Brazil). A final polishing was performed using felt discs and a 1-μm diamond suspension (ERIOS Equipamentos Eireli, São Paulo, Brazil).^22^

Three Knoop indentations were performed along a straight line from the center of the canal lumen toward the periphery of each specimen. Microhardness measurements were obtained before and after irrigation using a microhardness tester (Shimadzu Corporation, Tokyo, Japan) with a 25-g load applied for 15 seconds.^22^ Indentations were performed after treatments at the same locations as the baseline measurements. The mean of the three indentations was calculated and used for statistical comparison of microhardness changes before and after the irrigation protocols.

### Picrosirius red polarization method

After treatment, dentin blocks were immediately fixed in 10% buffered formalin. The specimens were washed in running tap water for 24 hours and subsequently decalcified in 10% EDTA prior to paraffin embedding. Serial 4-μm sections were obtained.

Histological sections were stained with Picrosirius Red for assessment of collagen content and organization following treatment. Under polarized light microscopy, collagen fibers exhibit birefringence, while non collagenous tissue components appear dark and are excluded from the analysis. Birefringence colors range from yellow-green (immature and thin fibers) to yellow-red (mature and thick fibers), thus reflecting differences in fiber thickness and organization.

Four central fields per specimen were analyzed using a polarized light microscope at ×20 magnification (Leica DM2700 P; Leica Microsystems, Wetzlar, Germany). The birefringent areas corresponding to green, yellow, and red collagen fibers, as well as the total birefringent area, were quantified using ImageJ software (version 1.36; National Institutes of Health, Bethesda, MD, USA) and expressed as pixels^23^. Color quantification was performed using a fixed hue threshold for each fiber color (green, yellow, and red), applied uniformly across all images by a single examiner blinded to group allocation.

### Statistical analysis

Data normality was assessed using the Shapiro-Wilk test. Picrosirius Red data showed non-normal distribution and were analyzed using the Kruskal-Wallis test followed by Dunn's post hoc test for intergroup comparisons and the Wilcoxon signed rank test for intragroup comparisons. OCT and microhardness data exhibited normal distribution and were analyzed using one way analysis of variance (ANOVA) followed by Tukey's multiple comparison test for intergroup comparisons, and the paired t-test for intragroup comparisons. Statistical significance was set at α = 0.05 (GraphPad Software; San Diego, USA).

## Results

A statistically significant increase was observed in irrigant diffusion after treatment for all experimental protocols (p < 0.05), except for CI with SS, which showed no significant difference compared with baseline values (p > 0.05). No differences among experimental groups (p > 0.05) were observed (Table). Representative OCT images illustrating irrigant diffusion patterns are shown in Figure 1.

**Table:** Mean and standard deviation (in parenthesis) for microhardness and optical coherence tomography analysis on the dentin surface before and after irrigation protocols: no activation (NA), conventional irrigation (CI), ultrasonic activation (UA), and EasyClean activation (EA).

Groups		Optical coherence tomography		Microhardness	
		Before	After	Before	After
Saline solution					
	NA	57.7 (± 16.7)^Aa^	89.5 (± 7.1)^Ba^	40.9 (± 6.5)^Aa^	45.4 (± 9.0)^Aa^
	CI	62.1 (± 16.5)^Aa^	78.5 (± 24.5)^Aa^	41.1 (± 5.7)^Aa^	36.6 (± 5.3)^Aa^
	UA	89.1 (± 17.8)^Aa^	11.8 (± 23.2)^Ba^	41.8 (± 6.3)^Aa^	46.2 (± 11.5)^Aa^
	EA	57.6 (± 19.9)^Aa^	84.0 (± 5.0)^Ba^	41.7 (± 5.7)^Aa^	38.5 (± 16.4)^Aa^
Sodium hypochlorite					
	NA	62.0 (± 16.1)^Aa^	105.4 (± 25.5)^Ba^	40.3 (± 7.8)^Aa^	35.0 (± 9.5)^Aa^
	CI	57.3 (± 11.6)^Aa^	89.5 (± 8.8)^Ba^	42.2 (± 6.6)^Aa^	47.7 (± 13.7)^Aa^
	UA	72.1 (± 23.1)^Aa^	88.3 (± 14.2)^Ba^	41.1 (± 7.1)^Aa^	41.7 (± 8.9)^Aa^
	EA	61.6 (± 22.8)^Aa^	81.3 (± 11.1)^Ba^	41.1 (± 6.3)^Aa^	45.9 (± 11.9)^Aa^
Chlorhexidine					
	NA	60.8 (± 22.7)^Aa^	76.7 (± 15.7)^Ba^	41.5 (± 7.3)^Aa^	37.8 (± 12.0)^Aa^
	CI	67.7 (± 15.9)^Aa^	80.3 (± 22.6)^Ba^	41.5 (± 6.6)^Aa^	40.4 (± 11.3)^Aa^
	UA	68.4 (± 17.5)^Aa^	98.0 (± 26.5)^Ba^	40.0 (± 4.8)^Aa^	43.3 (± 9.3)^Aa^
	EA	53.5 (± 10.9)^Aa^	86.9 (± 26.5)^Ba^	44.5 (± 12.4)^Aa^	39.4 (± 6.0)^Aa^

Different lowercase letters indicate statistically significant differences among groups (p < 0.05). Different uppercase letters indicate statistically significant differences between before and after treatment values within the same group (p < 0.05).

**Figure 1:**
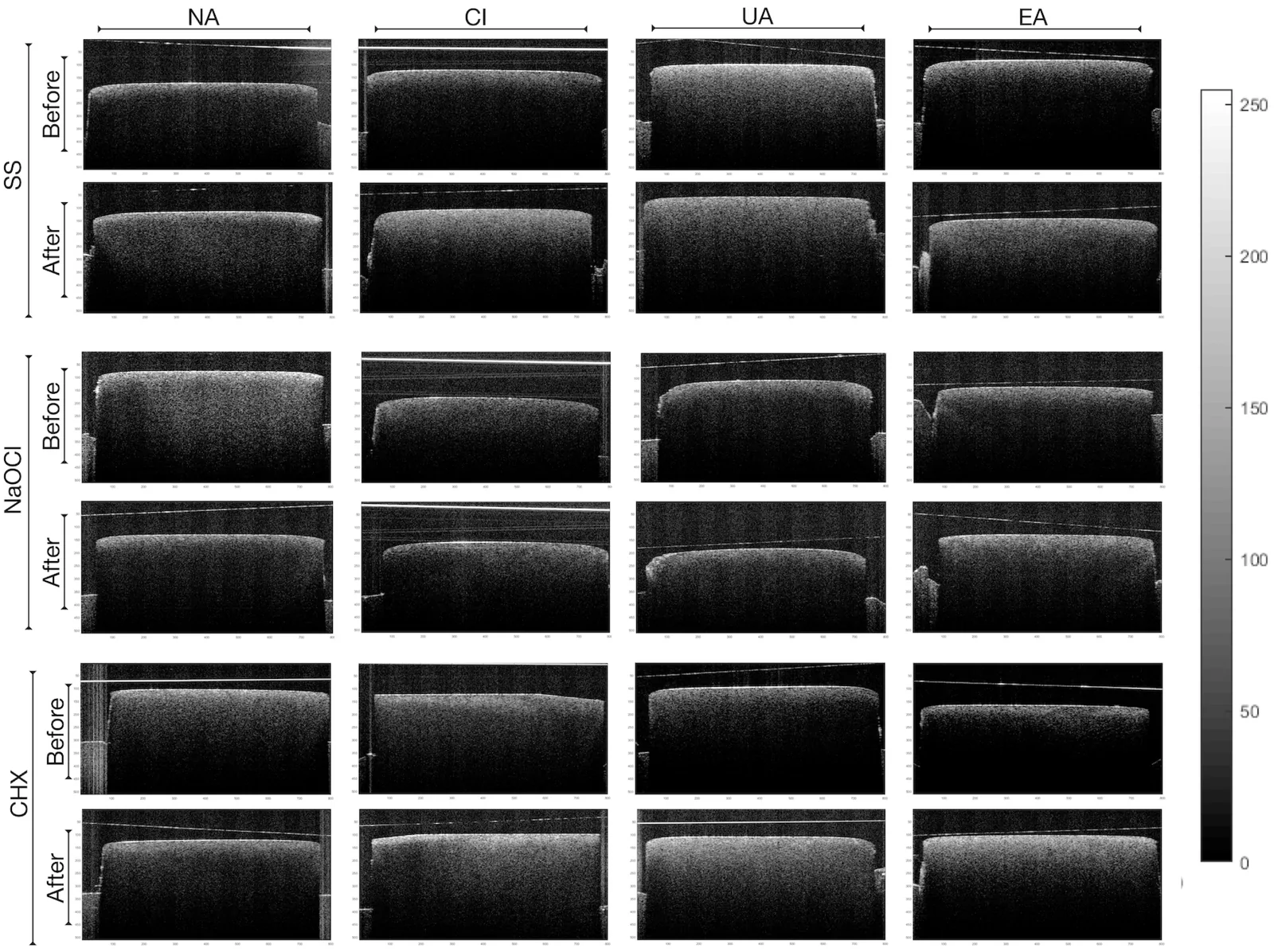
Representative optical coherence tomography (OCT) images of dentin slices obtained before and after irrigation with saline solution (SS), 2.5% sodium hypochlorite (NaOCl), and 2% chlorhexidine (CHX) under different irrigation protocols: no activation (NA), conventional irrigation (CI), ultrasonic activation (UA), and EasyClean activation (EA).

Microhardness analysis revealed no significant differences among experimental groups or between baseline and post-treatment values (p > 0.05) (Table).

The results of the Picrosirius Red analysis are presented in Figures 2 and 3. All experimental groups exhibited birefringent collagen fibers with green, yellow, and red coloration. Quantitative analysis of total birefringent area revealed no significant differences among groups (p > 0.05). Yellow fiber area was significantly different between specimens exposed to NaOCl and CHX for 30 minutes (NA) and EA with SS (p < 0.05). For red fibers, a significant difference was observed between the CHX group with NA and the SS with UA or 30-minute exposure (NA) (p < 0.05). In the analysis of green fibers, the CHX group with NA showed significant differences compared with CHX with UA and SS with NA, CI, and UA (p<0.05).

**Figure 2:**
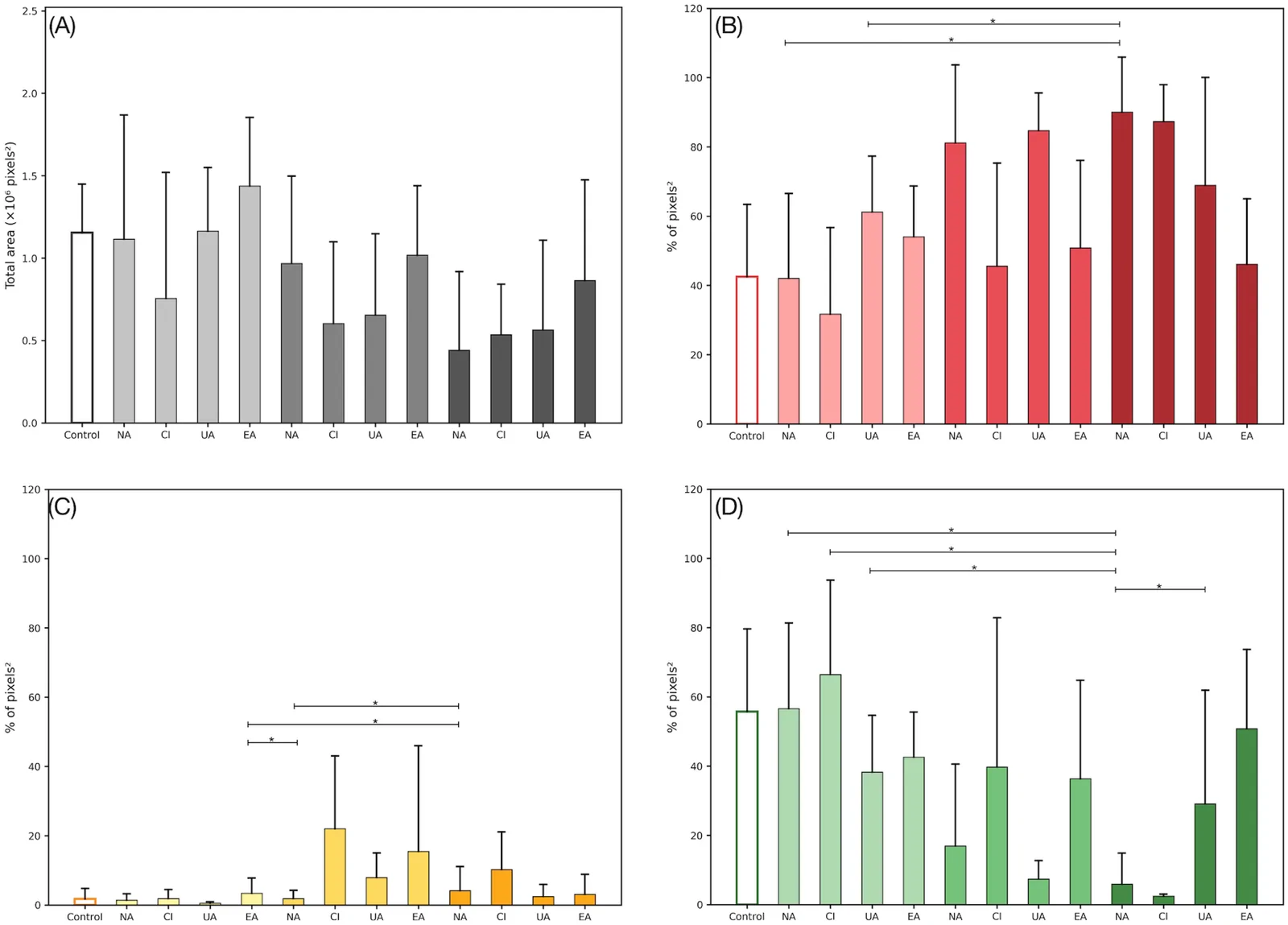
Quantitative analysis of dentin collagen by Picrosirius Red staining under polarized light microscopy. (A) Total birefringent area (×10⁵ pixels); (B) percentage of red fibers; (C) percentage of yellow fibers; and (D) percentage of green fibers relative to the total birefringent area. Specimens received no treatment (Control) or were irrigated with saline solution (SS), 2.5% sodium hypochlorite (NaOCl), or 2% chlorhexidine (CHX) and submitted to different irrigation protocols: no activation (NA), conventional irrigation (CI), ultrasonic activation (UA), and EasyClean activation (EA). Asterisks indicate statistically significant differences between groups (p < 0.05).

**Figure 3:**
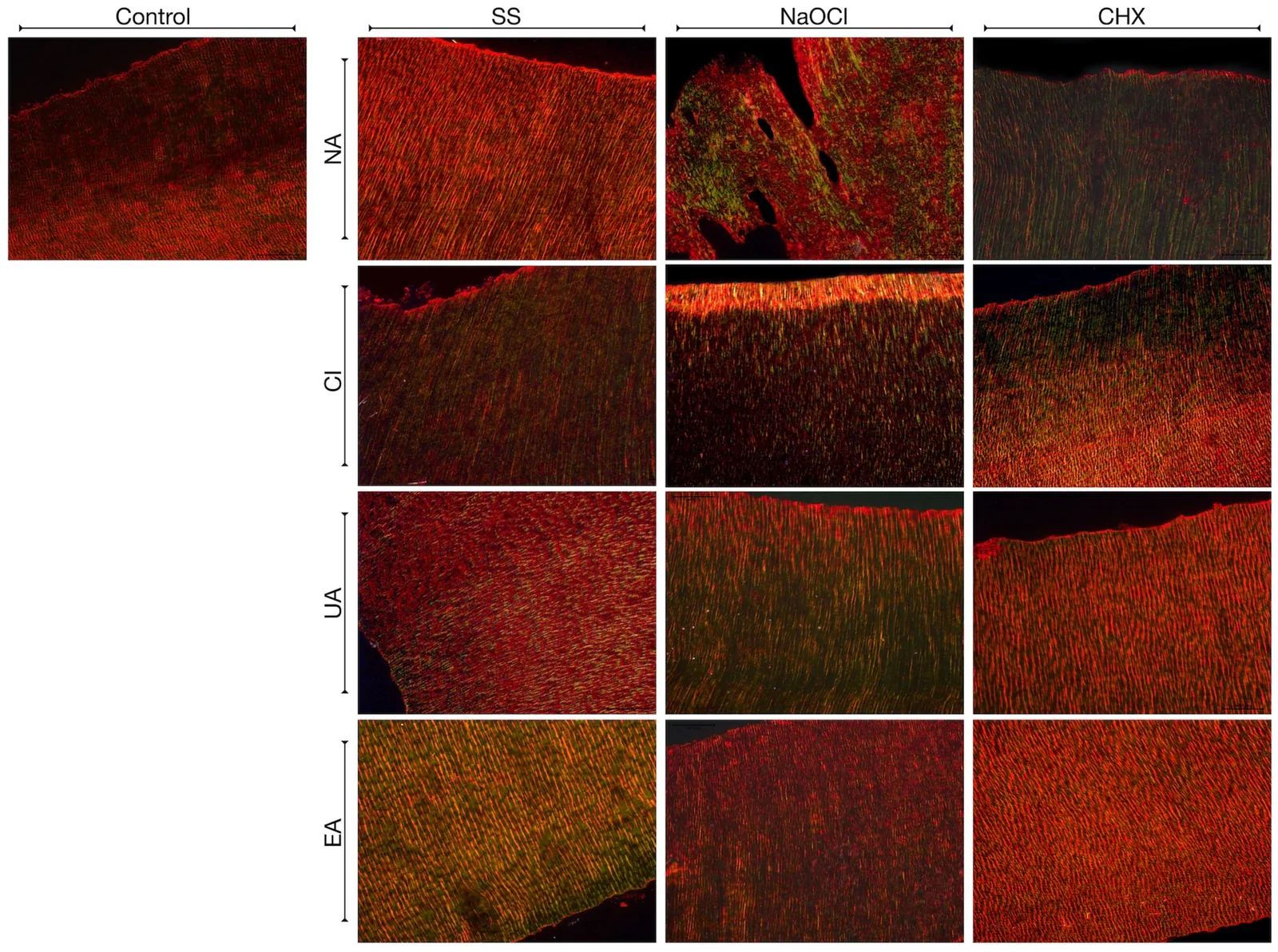
Representative polarized light microscopy images of dentin sections stained with Picrosirius Red after irrigation with saline solution (SS), 2.5% sodium hypochlorite (NaOCl), and 2% chlorhexidine (CHX), under different irrigation protocols: no activation (NA), conventional irrigation (CI), ultrasonic activation (UA), and EasyClean activation (EA).

## Discussion

This study aimed to evaluate the effects of different irrigation protocols on irrigant diffusion in dentin assessed by OCT, and their effects on dentin ultrastructure through microhardness testing and polarized light microscopy with Picrosirius Red staining. The results demonstrated that irrigation increased dentin diffusion compared with baseline in most experimental groups; however, no differences in diffusion or dentin microhardness were observed among irrigation protocols. In contrast, collagen fiber organization was influenced by the irrigant, contact time, and activation method. Therefore, the null hypothesis was partially rejected.

Optimizing irrigant diffusion is critical for achieving effective intracanal debridement and favorable long-term outcomes, but undesirable chemical alterations may also occur.^19^ Several experimental approaches have been used to investigate intracanal irrigant behavior, including histological sectioning, computational modeling, and imaging-based techniques.^7,24,25^ Within this context, OCT is a noninvasive imaging technique that allows high-resolution evaluation of dentin surface and subsurface microstructure.^14^ However, most studies evaluating irrigant flow using OCT have focused on its effects on biofilm behavior rather than on interactions with the dentin matrix.

In the present study, dentin diffusion increased after irrigation for all experimental protocols (p<0.05), except for conventional irrigation with saline solution. However, no differences were detected among groups (p > 0.05). Previous studies demonstrated that ultrasonic activation increased irrigant diffusion compared with conventional irrigation.^20^ However, irrigant diffusion is influenced not only by the activation method but also by root canal anatomy^26^ and the physical properties of the irrigating fluid, such as viscosity and flow behavior.^27^ Therefore, these factors should be considered when selecting the irrigation protocol in clinical practice.

The changes in dentin diffusion patterns detected by OCT after application of the irrigation protocols should be interpreted in light of the known interactions between the irrigating solutions and the dentin matrix. Chlorhexidine does not dissolve tissue and interacts with dentin primarily through electrostatic binding to the organic matrix, resulting in substantivity while causing minimal structural modification.^28,29^ Sodium hypochlorite, in contrast, interacts primarily with the organic component of dentin through proteolytic degradation of collagen and other noncollagenous proteins,^30^ which may increase dentin permeability and modify the optical scattering properties without necessarily inducing immediate mineral loss.^31^ These distinct interaction mechanisms highlight the importance of considering both the organic and inorganic aspects of dentin when interpreting the effects of irrigation protocols.

Microhardness testing measures dentin inorganic integrity by reflecting changes in mineral content and mechanical resistance, both of which contribute to the mechanical integrity and fracture resistance of endodontically treated teeth.^32,33^ In the present study, microhardness of superficial dentin adjacent to the root canal lumen was assessed using the Knoop indenter. Regardless of the agitation protocol, none of the irrigation solutions significantly affected dentin microhardness (p > 0.05). This finding is consistent with previous studies demonstrating that saline solution does not alter dentin properties and that NaOCl and CHX, when used alone at comparable concentrations, primarily interact with the organic component of dentin without inducing clinically relevant changes in its mineral-related mechanical properties.^16,22,33^ Although reductions in dentin microhardness have been reported with higher concentrations of NaOCl,^35^ clinically significant decreases are more common when these solutions are combined with chelating agents, which actively remove calcium ions from hydroxyapatite and promote dentin demineralization.^34^

Alterations in the collagen matrix directly affect its mechanical behavior, adhesive performance, and long-term tooth integrity. Type I collagen is the primary organic scaffold of dentin, serving as structural scaffold for mineral deposition and conferring toughness and fracture resistance through its hierarchical organization and cross-linking.^36^ Picrosirius Red staining under polarized light is a well-established method for assessing collagen content and organization.^15,23,37^ The shade of birefringence emitted by collagen fibers of different thicknesses can vary from green, indicating thinner and more dispersed fibers, to yellow and red, indicating gradually thicker, more organized, and compact fibers.^15,23,37^

In the present study, birefringent collagen fibers with green, yellow, and red coloration were observed in all experimental groups, indicating preservation of the collagen framework of dentin after irrigation. Quantitative analysis of the total birefringent area revealed no significant differences among groups, suggesting that none of the protocols induced a generalized loss of collagen content. However, analysis based on fiber color distribution revealed selective alterations associated with irrigant type, exposure time, and activation protocol.

Irrigation with saline solution resulted in a predominance of thick, mature collagen fibers, with no differences from the control group, regardless of exposure time or ultrasonic activation, confirming its minimal effect on organic dentin matrix.^2^ In contrast, prolonged contact with chlorhexidine resulted in a less organized collagen substrate and a higher proportion of immature fibers, an effect that was attenuated by passive ultrasonic agitation. These findings suggest that long exposure to CHX may interfere with collagen organization, potentially affecting the mechanical stability of dentin. Although CHX has been described as a matrix metalloproteinase inhibitor capable of preserving hybrid layers,^38^ its effects on dentinal collagen appear to depend strongly on exposure conditions and activation protocols.

NaOCl caused the disorganization of the collagen network only after prolonged exposure, while the overall birefringent fiber area remained comparable to the control group. As a strong oxidizing agent, NaOCl degrades the organic matrix through oxidation and chloramination reactions. Due to the small size of the hypochlorite ion, NaOCl can penetrate mineralized dentin and reach collagen fibrils, promoting their gradual degradation.^39^ The limited collagen disorganization observed in the present study may be attributed to the low NaOCl concentration and short exposure time, reinforcing the concentration- and time-dependent effects of NaOCl on dentinal collagen.^39^

This *in vitro* study has inherent limitations that should be considered when interpreting the findings. Although bovine dentin is widely used due to its structural similarity to human dentin, it does not fully replicate the biological complexity of the human root canal system, including variations in tubule density, dentinal fluid dynamics, and periapical buffering.^40^ In addition, the investigation focused on the immediate effects of irrigation protocols, without assessing long-term outcomes such as restoration durability or sustained changes in dentin mechanical properties. Moreover, while Picrosirius Red analysis, microhardness testing, and OCT provided objective structural information, functional outcomes such as bond strength or sealing ability were not evaluated. Future studies incorporating long-term assessments and clinically relevant functional endpoints are warranted to validate these findings.

## Conclusion

Within the limitations of this *in vitro* study, dentin diffusion increased similarly across the irrigation protocols, while dentin microhardness remained unchanged, indicating that neither outcome was significantly affected by irrigant type or activation method. In contrast, dentinal collagen organization was influenced by irrigant type, exposure time, and activation method. Prolonged contact with 2% CHX appeared more detrimental to collagen organization, whereas 2.5% NaOCl primarily altered dentin morphology without reducing mature collagen fiber content.

## Data Availability

The authors declare that all data generated or analyzed during this study are included in this published article.
